# Supplementary figures and images for: Clinical utility of inflammatory biomarkers in COVID-19 in direct comparison to other respiratory infections—A prospective cohort study
Source: PLoS One. 2022 May 27;17(5):e0269005. doi: 10.1371/journal.pone.0269005 (PMC9140295; doi:10.1371/journal.pone.0269005)

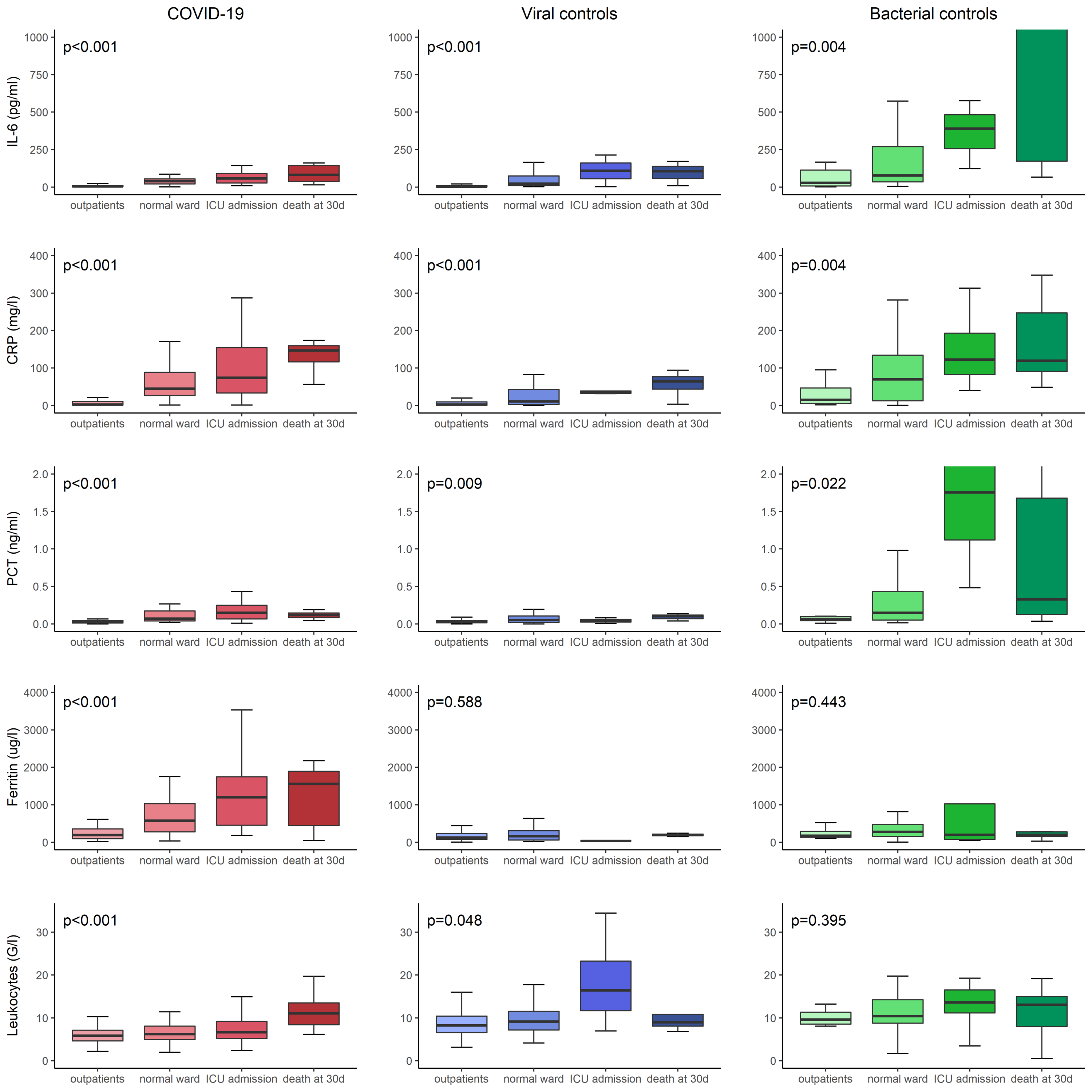

Supplement: S1 Fig — Disease severity is categorized in four categories; outpatients, normal ward, ICU admission, and death at 30 days; P-values were calculated using the Kruskal-Wallis test; COVID-19 = coronavirus disease 2019, IL-6 = interleukin-6, CRP = c-reactive protein, PCT = procalcitonin, ICU = intensive care unit. (TIF) [file pone.0269005.s001.tif]
