# Supplementary material for: Clinical utility of inflammatory biomarkers in COVID-19 in direct comparison to other respiratory infections—A prospective cohort study
Source: PLoS One. 2022 May 27;17(5):e0269005. doi: 10.1371/journal.pone.0269005 (PMC9140295; doi:10.1371/journal.pone.0269005)
Supplement: S2 Table — (DOCX) [file pone.0269005.s003.docx]

# Supporting information

## S2 Table. Missing values.
